# Supplementary material for: Resistance mechanisms of SARS-CoV-2 3CLpro to the non-covalent inhibitor WU-04
Source: Cell Discov. 2024 Apr 9;10:40. doi: 10.1038/s41421-024-00673-0 (PMC11003996; doi:10.1038/s41421-024-00673-0)
Supplement: Supplementary file 1 — Supplementary Information [file 41421_2024_673_MOESM1_ESM.pdf]

Supplementary Information for

Title: Resistance mechanisms of SARS-CoV-2 3CLpro to the non-covalent inhibitor WU-04

Authors: Lijing Zhang[1,2,3,4,8], Xuping Xie[5,8], Hannan Luo[2,3,4], Runtong Qian[2,3,4], Yang Yang[7], Hongtao Yu[2,3,4,6], Jing Huang[2,3,4], Pei-Yong Shi[5]*, Qi Hu[2,3,4]*

Affiliations:

[1]Zhejiang University, Hangzhou, Zhejiang, China.

[2]Westlake Laboratory of Life Sciences and Biomedicine, Hangzhou, Zhejiang, China.

[3]School of Life Sciences, Westlake University, Hangzhou, Zhejiang, China.

[4]Institute of Biology, Westlake Institute for Advanced Study, Hangzhou, Zhejiang, China.

[5]Department of Biochemistry and Molecular Biology, University of Texas Medical Branch, Galveston, TX, USA.

[6]Changping Laboratory, Yard 28, Science Park Road, Changping District, Beijing, China.

[7]Division of Life Sciences and Medicine, University of Science and Technology of China, Hefei, China.

[8]L.Z. and X.X. contributed equally to this work.

* Corresponding authors. Qi Hu, Pei-Yong Shi

Email: huqi@westlake.edu.cn; peshi@utmb.edu.

a

WU-04-resistant mutations in 3CLpro

Viruses	EC_{50}s (nM)	3CL mutations
WT mNG	18.2, 17.2	NA
14h6 P7-1	> 5000	T10200A (3CL: M49K), A10547G (3CL: M165V)
14h6 P7-2	> 5000	T10200A (3CL: M49K), A10547G (3CL: M165V)
14h6 P7-3	> 5000	T10200A (3CL: M49K), T10955C (3CL: S301P)
14h6 P7-4	> 5000	T10200A (3CL: M49K), T10955C (3CL: S301P)

b

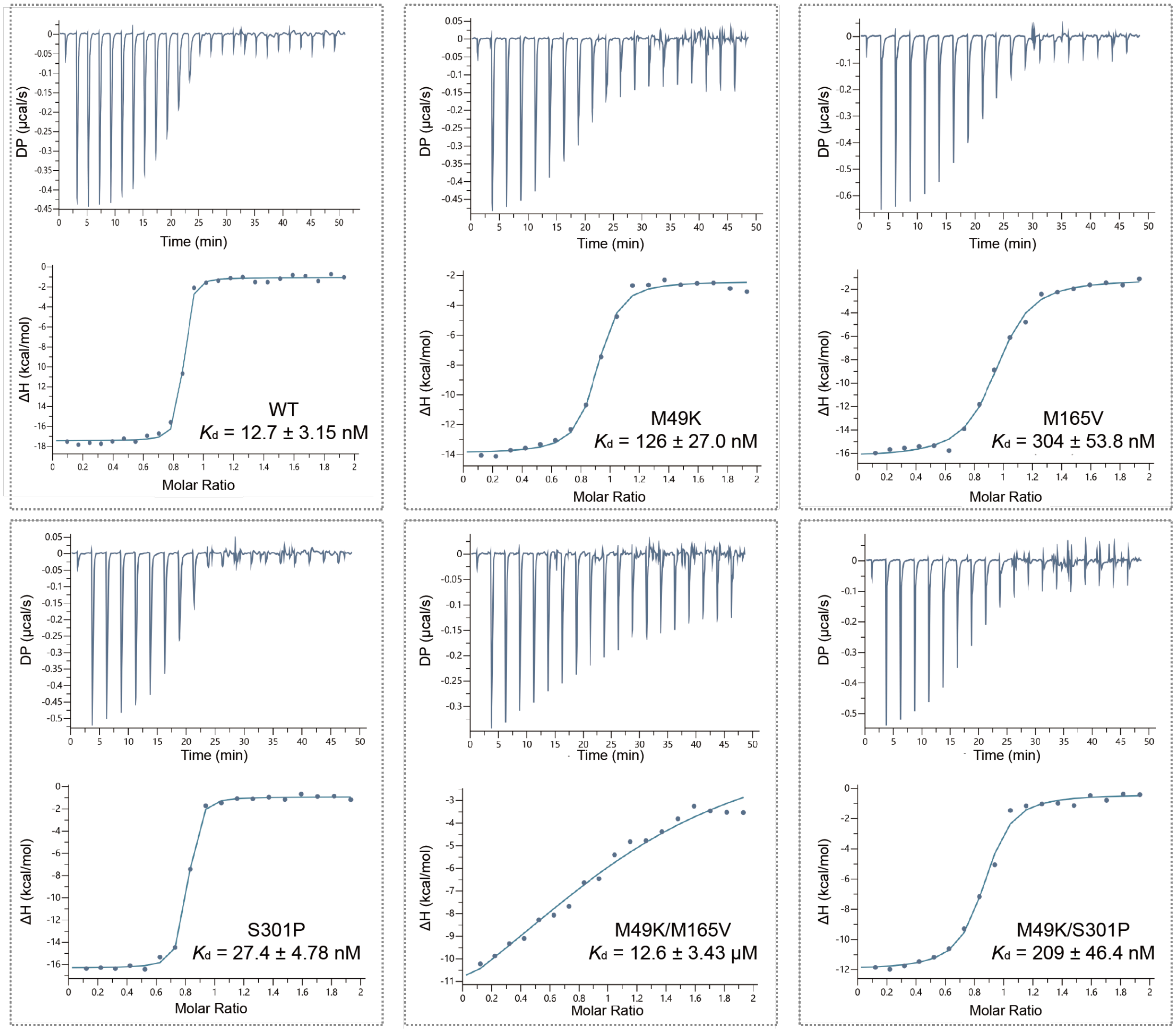

Supplementary Fig. S1 Identification of WU-04-resistant mutations in SARS-CoV-2 3CLpro. a The WU-04-resistant viruses were identified by serial passaging of the SARS-CoV-2

mNG in Vero E6 cells treated with increasing concentrations of WU-04. **b** The binding affinities (K_d) between WU-04 and the WU-04-resistant 3CLpro mutants were measured using isothermal titration calorimetry (ITC).

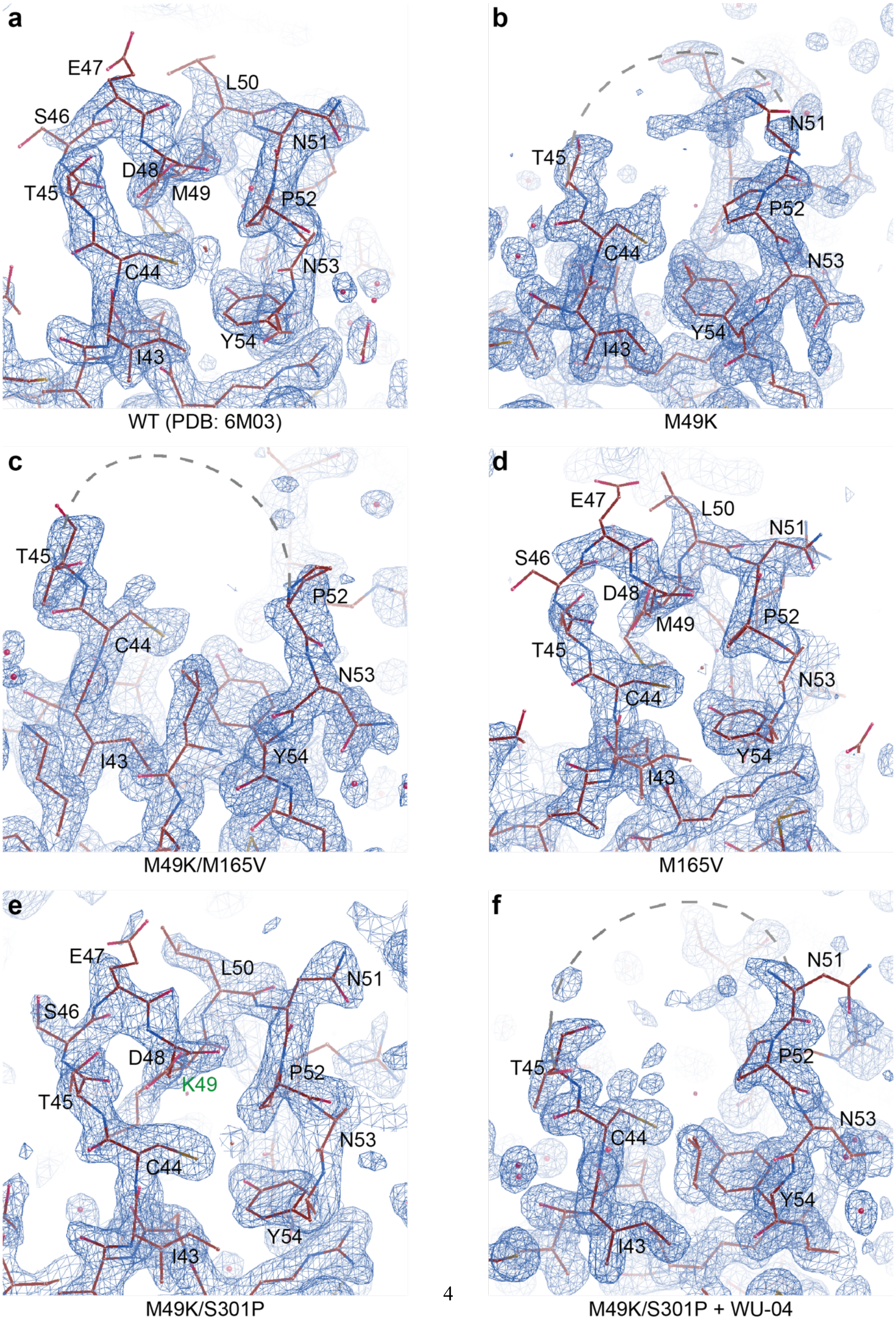

a
E47
L50
S46
N51
D48
M49
T45
P52
C44
N53
Y54
I43
WT (PDB: 6M03)
b
N51
T45
P52
C44
N53
Y54
I43
M49K
c
T45
P52
C44
N53
I43
Y54
M49K/M165V
d
E47
L50
N51
S46
D48
M49
P52
T45
N53
C44
Y54
I43
M165V
e
E47
L50
N51
S46
D48
K49
P52
T45
N53
C44
Y54
I43
M49K/S301P
f
N51
P52
T45
N53
C44
Y54
I43
M49K/S301P + WU-04

Supplementary Fig. S2 The *2mFo-DFc* electron density maps of the helix (residues 45–51) of the crystal structures. The *2mFo-DFc* electron density maps of the helix (residues 45–51) of the wild-type SARS-CoV-2 3CLpro (PDB code: 6M03) (**a**), the WU-04-resistant mutants M49K (**b**), M49K/M165V (**c**), M165V (**d**), M49K/S301P (**e**), and the M49K/S301P mutant in complex with WU-04 (**f**) were contoured at a level of 1.2 RMSD in *coot*.

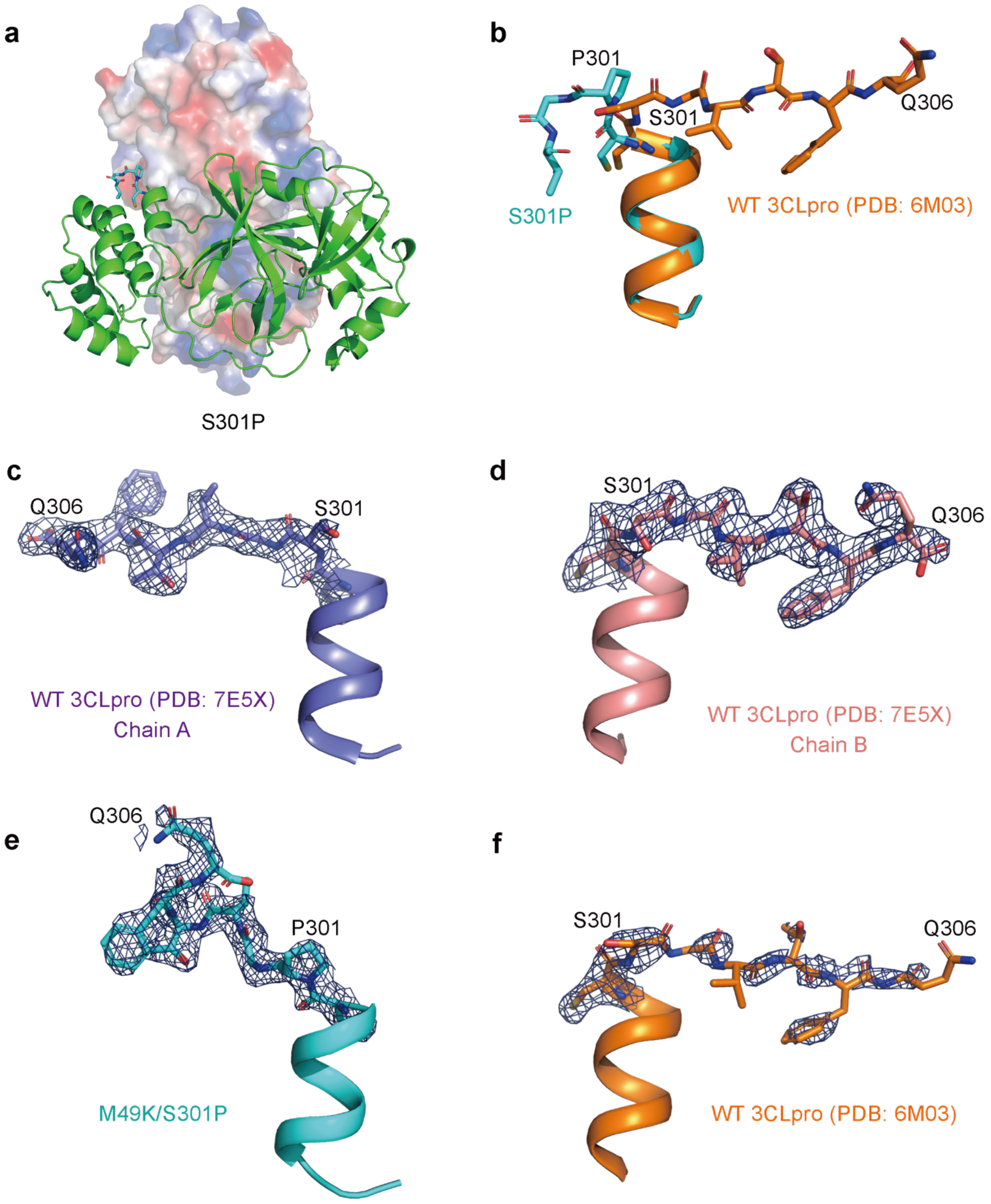

Supplementary Fig. S3 The C-terminal tails (residues 301–306) of 3CLpro in the crystal

structures. a In the crystal structure of the S301P mutant, the C-terminal tail (colored cyan) of one 3CLpro protomer turns away from the other 3CLpro protomer within the same 3CLpro homodimer. **b** Alignment of the C-terminal tails of 3CLpro in the S301P structure (colored cyan) with that in the mature WT 3CLpro structure (colored orange). **c-f** The *2Fo-Fc* maps of the C-terminal tails (residues 301–306) of the WT 3CLpro in the post-cleavage state (PDB code: 7E5X) (**c**, **d**), in the crystal structure of the WU-04 04-resistant mutant M49K/S301P (**e**), and that in the crystal structure of the WT 3CLpro in the mature state (PDB code: 6M03) (**f**) were made using PyMOL. The contour level was 1.0 σ.

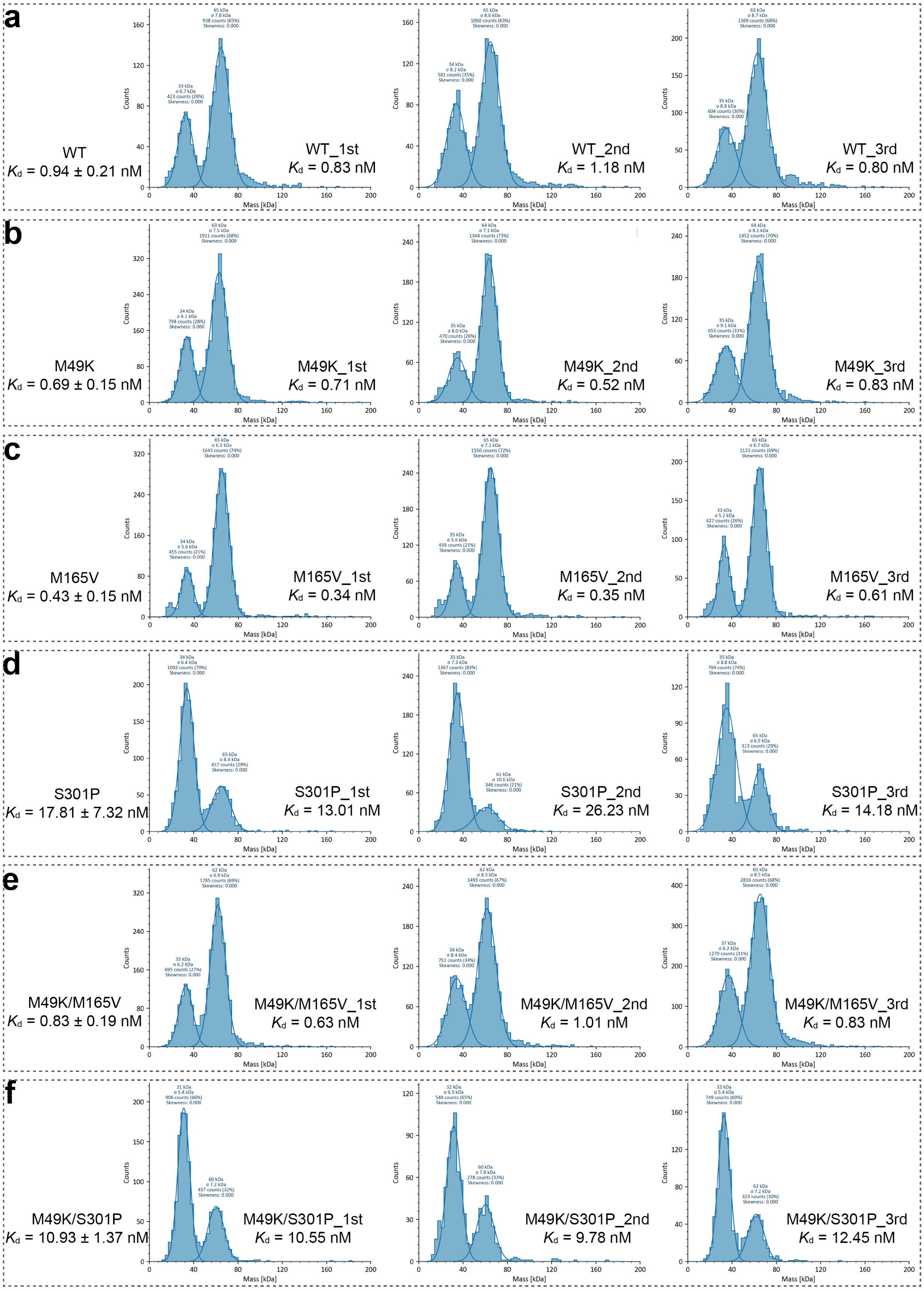
a
WT
K_d = 0.94 ± 0.21 nM
WT_1st
K_d = 0.83 nM
WT_2nd
K_d = 1.18 nM
WT_3rd
K_d = 0.80 nM
b
M49K
K_d = 0.69 ± 0.15 nM
M49K_1st
K_d = 0.71 nM
M49K_2nd
K_d = 0.52 nM
M49K_3rd
K_d = 0.83 nM
c
M165V
K_d = 0.43 ± 0.15 nM
M165V_1st
K_d = 0.34 nM
M165V_2nd
K_d = 0.35 nM
M165V_3rd
K_d = 0.61 nM
d
S301P
K_d = 17.81 ± 7.32 nM
S301P_1st
K_d = 13.01 nM
S301P_2nd
K_d = 26.23 nM
S301P_3rd
K_d = 14.18 nM
e
M49K/M165V
K_d = 0.83 ± 0.19 nM
M49K/M165V_1st
K_d = 0.63 nM
M49K/M165V_2nd
K_d = 1.01 nM
M49K/M165V_3rd
K_d = 0.83 nM
f
M49K/S301P
K_d = 10.93 ± 1.37 nM
M49K/S301P_1st
K_d = 10.55 nM
M49K/S301P_2nd
K_d = 9.78 nM
M49K/S301P_3rd
K_d = 12.45 nM
Counts
Mass [kDa]

Supplementary Fig. S4 Measurement of the dissociation constant (*K_d*) values for the WT and mutant 3CLpro to form homodimers. The K_d values of the SARS-CoV-2 WT 3CLpro (**a**), and the M49K (**b**), M165V (**c**), S301P (**d**), M49K/M165V (**e**) and M49K/S301P (**f**) mutants were determined using mass photometry. The data were processed and analyzed by Refeyn DiscoverMP. The Gaussian curves were fitted to each histogram distribution to determine the mass (kDa) and normalized counts. Three independent measurements were carried out to calculate the K_d value (mean ± SD) of each protein.

a

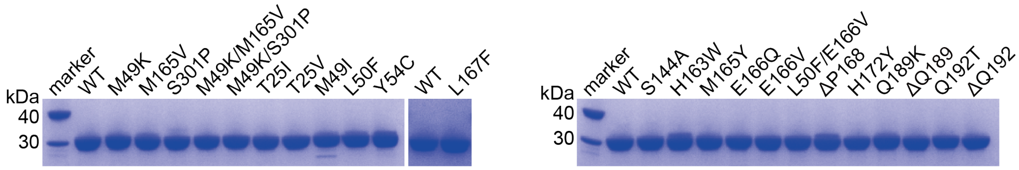

b

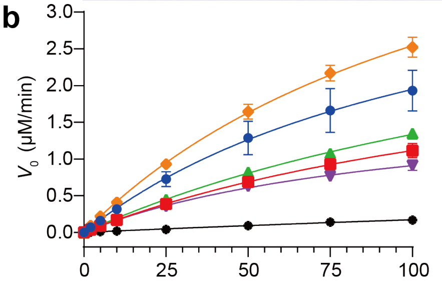

	V_{max} (μM·min^{-1})	K_m (μM)	k_{cat} (min^{-1})	k_{cat}/K_m (μM^{-1}·min^{-1})
WT	4.26 ± 0.83	119 ± 37	170.4	1.43
T25I	2.94 ± 0.33	163 ± 27	117.6	0.72
T25V	3.87 ± 0.27	190 ± 18	154.8	0.81
M49I	1.81 ± 0.13	100 ± 12	72.4	0.73
L50F	5.78 ± 0.41	127 ± 14	231.2	1.82
Y54C	1.29 ± 0.83	651 ± 469	51.6	0.08

c

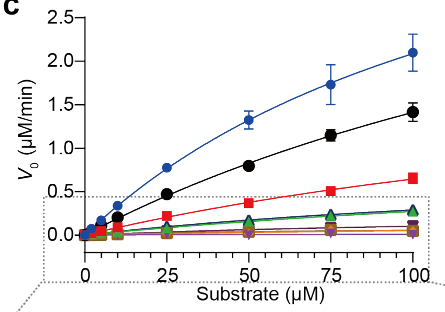

	V_{max} (μM·min^{-1})	K_m (μM)	k_{cat} (min^{-1})	k_{cat}/K_m (μM^{-1}·min^{-1})
WT	4.94 ± 0.71	137 ± 30	197.6	1.44
S144A	2.25 ± 0.40	249 ± 58	90	0.36
E166Q	1.03 ± 0.36	281 ± 123	41.2	0.15
E166V	ND	ND	ND	ND
L50F/E166V	ND	ND	ND	ND
ΔP168	4.83 ± 0.78	242 ± 52	193.2	0.8
H172Y	ND	ND	ND	ND
Q189K	0.86 ± 0.12	196 ± 38	34.24	0.17
Q192T	0.28 ± 0.18	172 ± 160	11.16	0.06

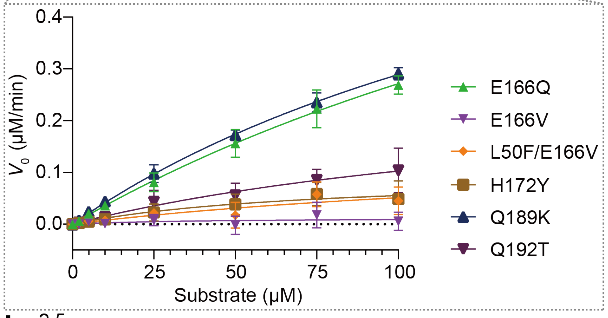

d

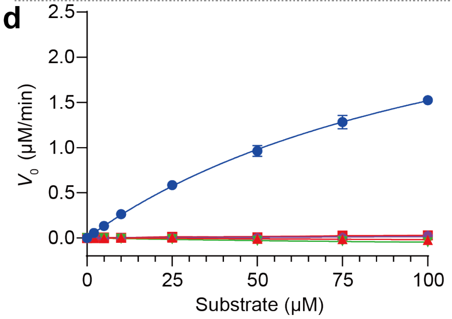

	V_{max} (μM·min^{-1})	K_m (μM)	k_{cat} (min^{-1})	k_{cat}/K_m (μM^{-1}·min^{-1})
WT	3.38 ± 0.21	122 ± 12	135.2	1.11
H163W	ND	ND	ND	ND
M165Y	ND	ND	ND	ND
ΔQ189	ND	ND	ND	ND
ΔQ192	ND	ND	ND	ND

e

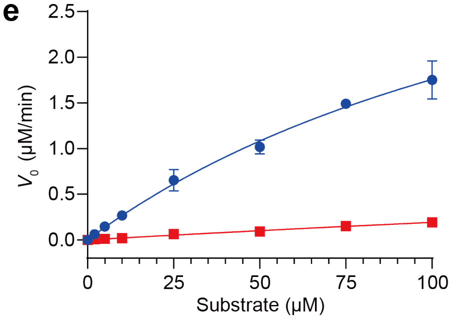

	V_{max} (μM·min^{-1})	K_m (μM)	k_{cat} (min^{-1})	k_{cat}/K_m (μM^{-1}·min^{-1})
WT	4.66 ± 0.78	165 ± 40	186.4	1.13
L167F	1.89 ± 2.93	886 ± 1499	75.6	0.09

Supplementary Fig. S5 The enzymatic activities of SARS-CoV-2 3CLpro mutants. The enzymatic activity of each mutant was evaluated using a FRET-based assay. The data of WT 3CLpro used in Supplementary Fig. S5b was also used in Fig. 1d. The data represent the mean ± SD of three independent measurements.

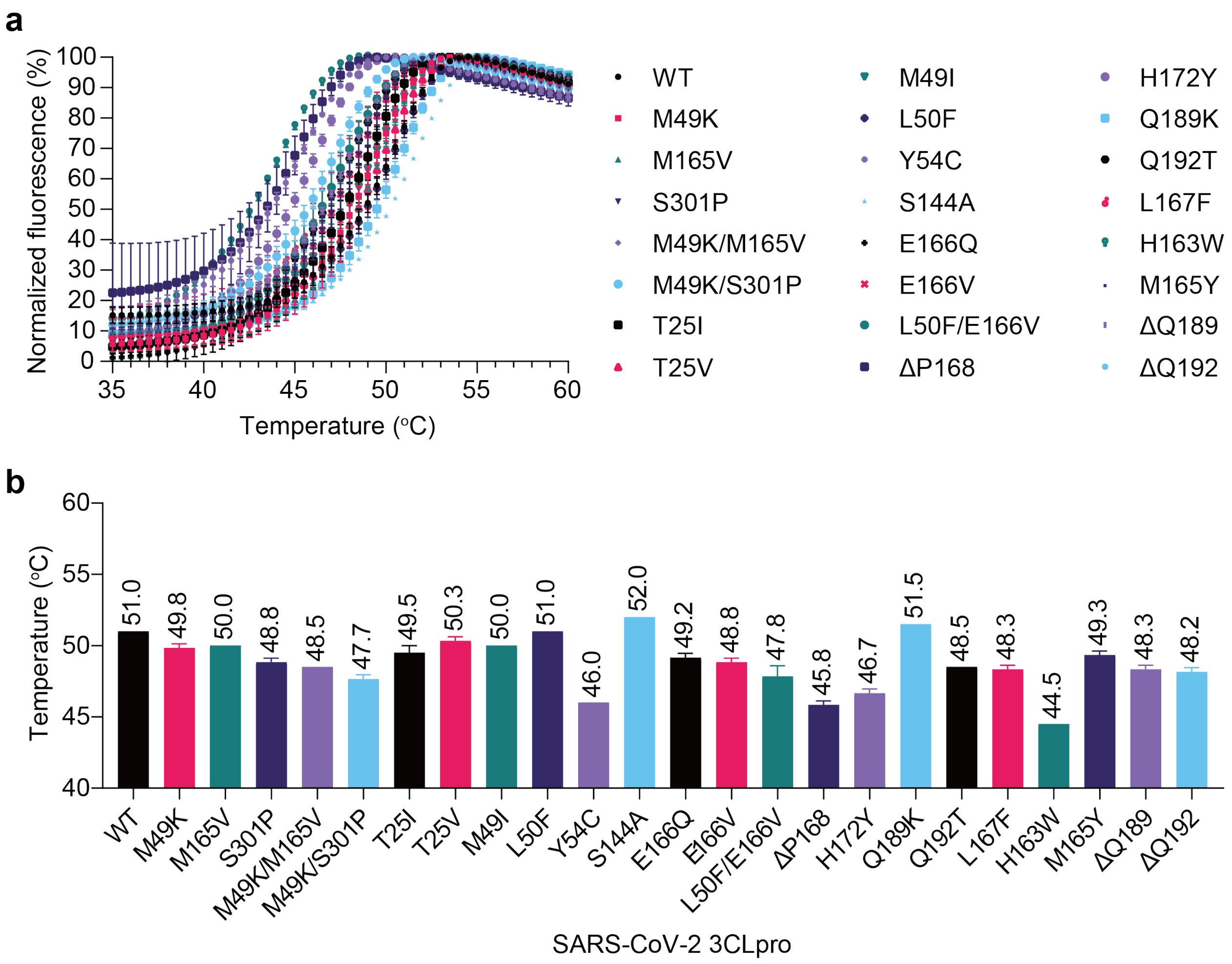

Supplementary Fig. S6 Thermal shift results of the SARS-CoV-2 3CLpro mutants. a The melting curves of the WT 3CLpro and the drug-resistant mutants. **b** The calculated melting temperatures of the WT 3CLpro and the drug-resistant mutants. The data represent the mean ± SD of technical triplicate.

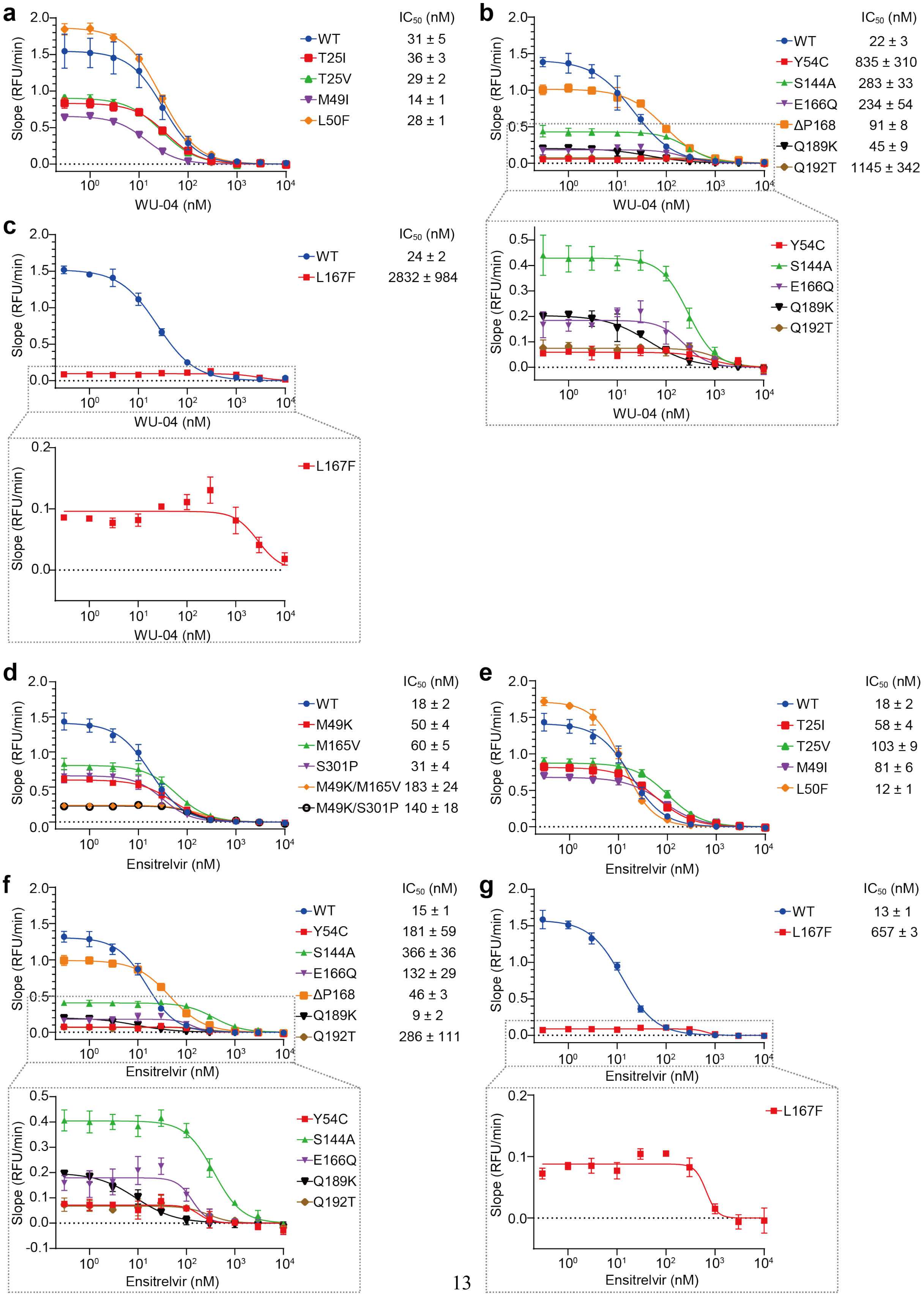
a
IC50 (nM)
WT 31 ± 5
T25I 36 ± 3
T25V 29 ± 2
M49I 14 ± 1
L50F 28 ± 1
Slope (RFU/min)
WU-04 (nM)
b
IC50 (nM)
WT 22 ± 3
Y54C 835 ± 310
S144A 283 ± 33
E166Q 234 ± 54
ΔP168 91 ± 8
Q189K 45 ± 9
Q192T 1145 ± 342
Slope (RFU/min)
WU-04 (nM)
Y54C
S144A
E166Q
Q189K
Q192T
c
IC50 (nM)
WT 24 ± 2
L167F 2832 ± 984
Slope (RFU/min)
WU-04 (nM)
L167F
d
IC50 (nM)
WT 18 ± 2
M49K 50 ± 4
M165V 60 ± 5
S301P 31 ± 4
M49K/M165V 183 ± 24
M49K/S301P 140 ± 18
Slope (RFU/min)
Ensitrelvir (nM)
e
IC50 (nM)
WT 18 ± 2
T25I 58 ± 4
T25V 103 ± 9
M49I 81 ± 6
L50F 12 ± 1
Slope (RFU/min)
Ensitrelvir (nM)
f
IC50 (nM)
WT 15 ± 1
Y54C 181 ± 59
S144A 366 ± 36
E166Q 132 ± 29
ΔP168 46 ± 3
Q189K 9 ± 2
Q192T 286 ± 111
Slope (RFU/min)
Ensitrelvir (nM)
Y54C
S144A
E166Q
Q189K
Q192T
g
IC50 (nM)
WT 13 ± 1
L167F 657 ± 3
Slope (RFU/min)
Ensitrelvir (nM)
L167F

Supplementary Fig. S7 The inhibitory activities (IC_{50}) of WU-04 (a-c) and Ensitrelvir (d-g) against 3CLpro were evaluated using a FRET-based assay. The data represent the mean ± SD of three independent measurements. The data of the WT 3CLpro in Supplementary Fig. S7a and S7d are also used in Fig. 1b and S7e, respectively.

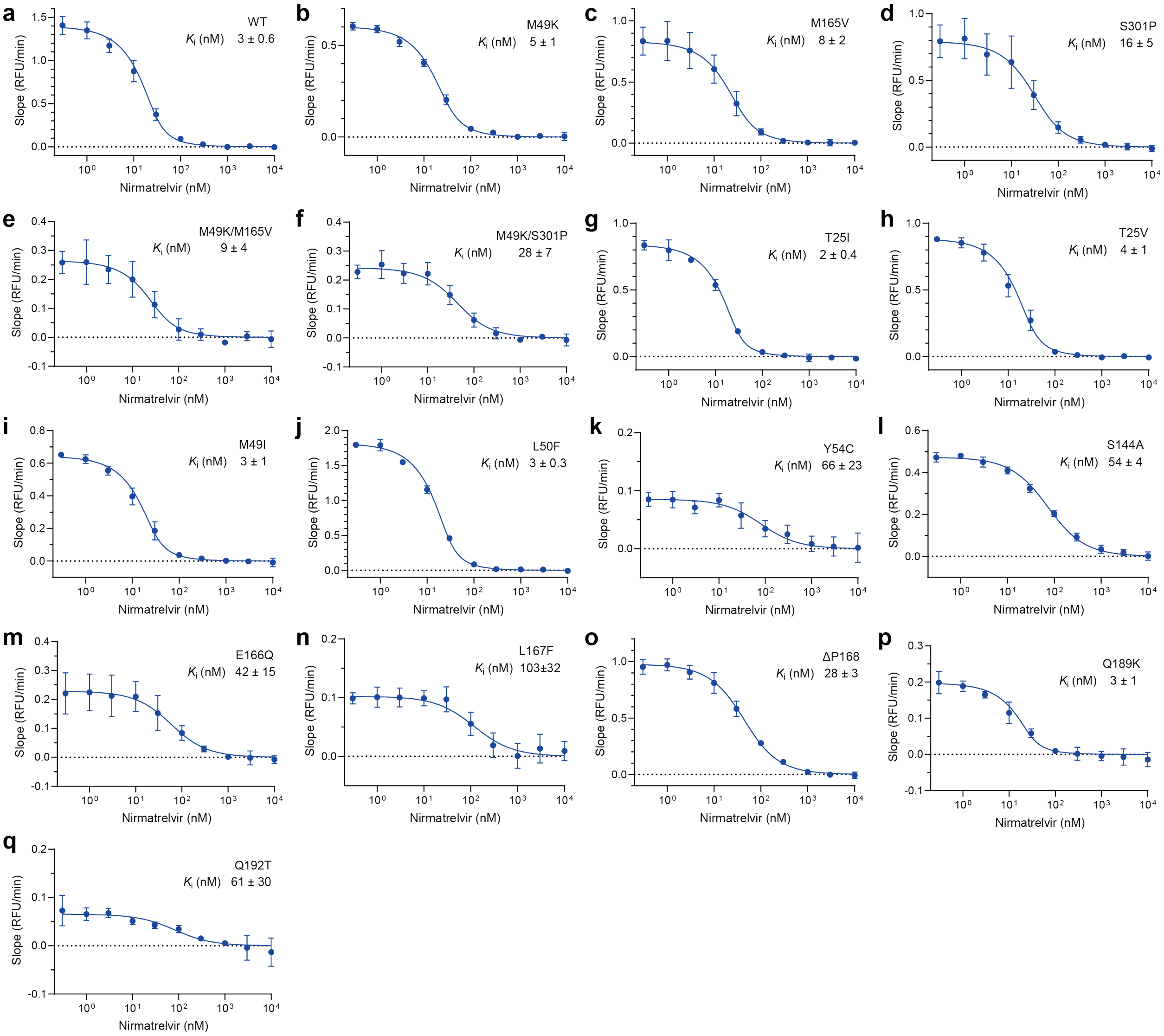

Supplementary Fig. S8 The inhibitory constants (*K*i) of nirmatrelvir against the WT 3CLpro (a) and the drug-resistant mutants (b-q) were evaluated using a FRET-based assay. The data represent the mean ± SD of three independent measurements.

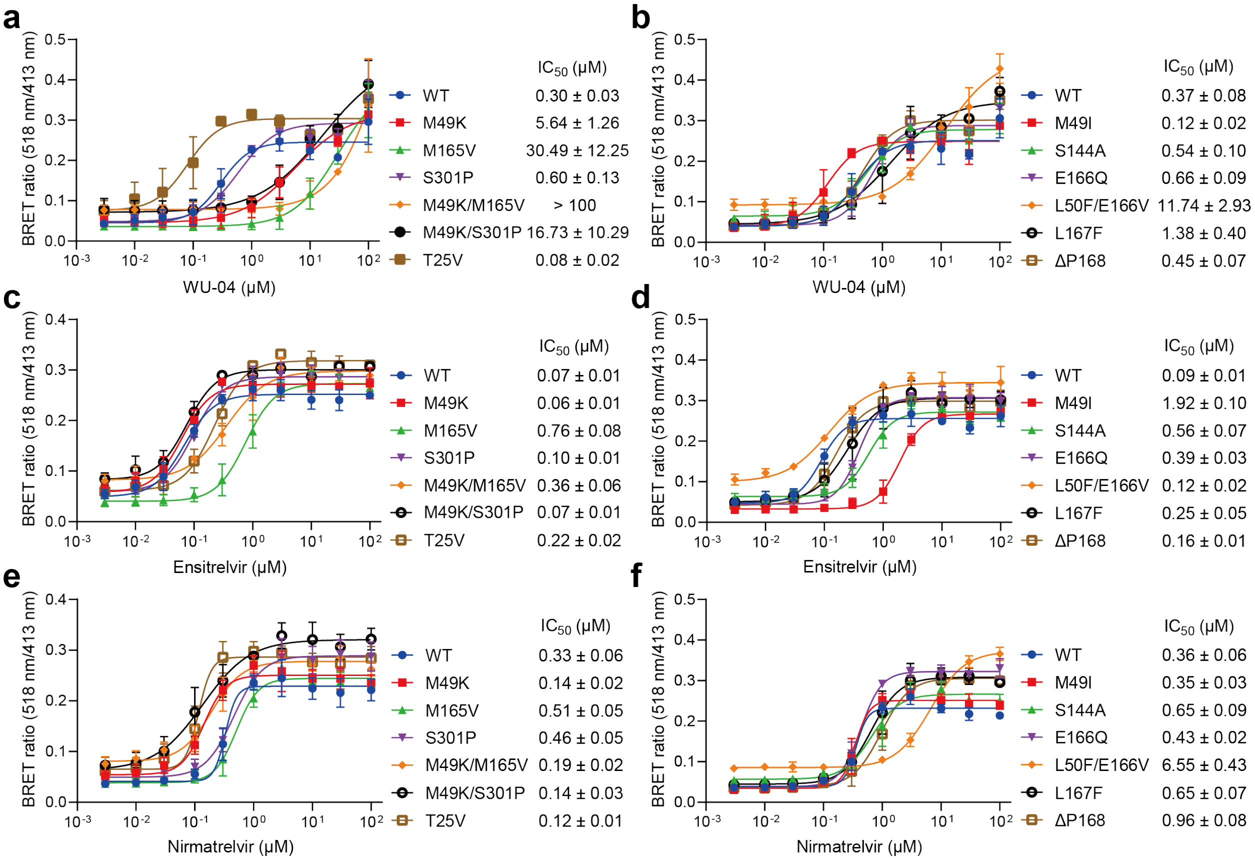

Supplementary Fig. S9 The inhibitory activities of WU-04 (**a**, **b**), ensitrelvir (**c**, **d**) and nirmatrelvir (**e**, **f**) against 3CLpro were evaluated by a BRET-based assay in HEK 293T cells. The data represent the mean ± SD of three independent measurements.

Supplementary Table S1. 3CLpro (NSP5) mutation counts in the GISAID database.

Position	WT (counts)	Mutant (counts)
49	M (15548971)	K (24); I (2344)
165	M (15546456)	V (18); Y (4758)
301	S (15550494)	P (22)
25	T (15550539)	I (1043); V (1)
50	L (15546092)	F (5328)
54	Y (15551450)	C (12)
144	S (15551564)	A (19)
166	E (15546519)	Q (4764); V (40)
167	L (15551296)	F (21)
168	P (15550992)	deletion (161)
172	H (15551528)	Y (26)
189	Q (15550231)	deletion (1192); K (183)
192	Q (15550004)	deletion (1314); T (221)

Supplementary Table S2. Data collection and refinement statistics (statistics for the highest-resolution shell are shown in parentheses)

	M49K	*M165V*	*S301P*	*M49K/M165V*	*M49K/S301P*	*M49K/S301P+ WU-04*
Wavelength	1.541	1.541	1.541	1.541	1.541	1.541
Resolution range	29.66 - 1.5 (1.554 - 1.5)	28.01 - 2.2 (2.279 - 2.2)	27.87 - 2.0 (2.072 - 2.0)	29.63 - 1.5 (1.554 - 1.5)	29.56 - 2.21 (2.289 - 2.21)	30 - 1.65 (1.709 - 1.65)
Space group	I 1 2 1	I 1 2 1	I 1 2 1	I 1 2 1	P 1 21 1	I 1 2 1
Cell dimension a, b, c (Å)	51.5257, 81.6172, 90.2145	44.6974, 53.6278, 114.183	44.6192, 53.4037, 113.384	51.582, 80.1127, 90.3169	48.8593, 107.074, 54.261	51.5706, 81.3544, 89.5668
Cell dimension α，β，γ (°)	90, 96.9412, 90	90, 101.126, 90	90, 100.529, 90	90, 96.8662, 90	90, 103.666, 90	90, 97.3029, 90
Asymmetric unit	1	1	1	1	2	1
Total reflections	600407 (37929)	45420 (4516)	86547 (8225)	592321 (36637)	159440 (16557)	464381 (28789)
Unique reflections	59233 (5920)	13558 (1331)	17820 (1750)	58288 (5781)	27130 (2692)	44111 (4387)
Multiplicity	10.1 (6.4)	3.4 (3.4)	4.9 (4.7)	10.2 (6.3)	5.9 (6.2)	10.5 (6.6)
Completeness (%)	99.57 (99.41)	99.39 (98.95)	99.65 (99.60)	99.93 (99.53)	99.77 (100.00)	99.98 (100.00)
Mean I/sigma(I)	24.16 (1.68)	10.53 (2.89)	14.69 (3.85)	28.44 (1.57)	16.83 (4.56)	33.21 (6.49)
Wilson B-factor	15.78	31.49	24.66	16.16	26.40	12.78
R-merge	0.06024 (1.044)	0.1404 (0.4566)	0.08929 (0.3017)	0.04897 (1.12)	0.1172 (0.3458)	0.05714 (0.2446)
R-meas	0.06296 (1.135)	0.1661 (0.5359)	0.1006 (0.3394)	0.05135 (1.22)	0.1289 (0.3775)	0.05971 (0.2658)
R-pim	0.0179 (0.436)	0.08743 (0.2766)	0.04548 (0.1533)	0.01508 (0.4758)	0.05306 (0.1502)	0.01704 (0.1029)
CC1/2	1 (0.869)	0.968 (0.337)	0.996 (0.906)	1 (0.749)	0.994 (0.914)	0.999 (0.976)
*CC**	1 (0.964)	0.992 (0.71)	0.999 (0.975)	1 (0.925)	0.999 (0.977)	1 (0.994)
Reflections used in refinement	59016 (5912)	13528 (1323)	17819 (1750)	58283 (5779)	27111 (2692)	44109 (4387)
Reflections used for R-free	1993 (199)	1355 (133)	1782 (175)	2000 (198)	1482 (147)	1825 (182)
R-work	0.1942 (0.2494)	0.2084 (0.2670)	0.1792 (0.1995)	0.1961 (0.2700)	0.2281 (0.2830)	0.1626 (0.1607)
R-free	0.2053 (0.2942)	0.2511 (0.3048)	0.2352 (0.2833)	0.2084 (0.2703)	0.2806 (0.3522)	0.1810 (0.2080)
CC (work)	0.963 (0.910)	0.956 (0.803)	0.966 (0.922)	0.964 (0.863)	0.942 (0.847)	0.966 (0.950)
CC (free)	0.967 (0.815)	0.944 (0.637)	0.949 (0.847)	0.959 (0.854)	0.890 (0.786)	0.964 (0.935)
Number of non-hydrogen atoms	2548	2422	2435	2529	4841	2681
macromolecules	2290	2367	2341	2299	4740	2291
ligands	0	0	0	0	0	34
solvent	258	55	94	230	101	356
Protein residues	296	306	303	298	612	296
RMS (bonds)	0.009	0.002	0.008	0.006	0.002	0.009
RMS (angles)	1.17	0.46	0.94	0.84	0.54	1.11

Ramachandran favored (%)	98.63	96.71	97.67	96.94	96.88	98.63
Ramachandran allowed (%)	1.37	3.29	1.99	3.06	3.12	1.37
Ramachandran outliers (%)	0.00	0.00	0.33	0.00	0.00	0.00
Rotamer outliers (%)	0.00	0.38	0.77	0.00	0.57	0.00
Clashscore	1.54	1.50	2.59	1.32	3.73	1.53
Average B-factor	23.95	40.10	28.87	23.27	33.23	17.79
macromolecules	23.11	40.19	28.81	22.48	33.34	16.35
ligands						14.19
solvent	31.42	36.38	30.49	31.22	28.44	27.36
Number of TLS groups	1	1	1		1	1
